# Intrapersonal and Interpersonal Factors Promoting Posttraumatic Growth: A Longitudinal Study Immediately After Traumatic Loss

**DOI:** 10.1002/cpp.70174

**Published:** 2025-11-19

**Authors:** Philipp Jann, Jennifer Gräfenstein, Tobias Hecker

**Affiliations:** ^1^ Department of Clinical Psychology & Violence Research Bielefeld University Bielefeld Germany; ^2^ Institute for Emergency Psychology Bielefeld Germany; ^3^ Institute for Interdisciplinary Research on Conflict and Violence Bielefeld University Bielefeld Germany

**Keywords:** cognitions, immediately, longitudinal, loss, posttraumatic growth, social support

## Abstract

**Background:**

In response to extreme life events, individuals may experience not only distress but also positive transformation, known as posttraumatic growth (PTG). Only a few studies have investigated PTG following traumatic loss. This is the first study to investigate promotive factors—loss‐related, intrapersonal and interpersonal factors—immediately after the loss and 6 months later.

**Methods:**

A total of 36 participants (58% female, *Mdn* = 49 years) were invited by psychosocial crisis intervention teams. Data collection took place immediately after a traumatic loss (T1) and 6 months later (T2). Various sociodemographic, loss‐related, intrapersonal (e.g., symptomatic distress, posttraumatic cognitions) and interpersonal (e.g., social acknowledgement, self‐disclosure) variables were investigated. Sociodemographic and loss‐related variables were compared using inferential statistical group comparisons with regard to PTG. Multiple logistic regressions compared intrapersonal versus interpersonal factors to predict PTG at 6 months.

**Results:**

Participants were strongly affected by their level of exposure and the sudden or violent death circumstances of their close relatives. Among the sociodemographic and loss‐related characteristics, no association was found with PTG. Lower levels of acute symptomatic distress predicted higher PTG. After 6 months, interpersonal factors significantly predicted PTG, whereas intrapersonal variables showed no more association with PTG.

**Conclusions:**

The results suggest that there is a complex relationship between intrapersonal and interpersonal factors and PTG immediately following traumatic losses as well as 6 months later, which can only partially be related to the existing literature. Further research in this important field is urgently needed to support individuals after traumatic losses.

## Summary

- Intrapersonal and interpersonal factors are central to the development of posttraumatic growth (PTG).
- We found no evidence that sociodemographic or loss‐related variables are associated with differences in PTG following traumatic loss.
- We found indications, relevant for psychosocial crisis intervention teams, that lower initial levels of symptomatic distress may predict higher PTG 6 months after the loss.
- For clinical practitioners, the data suggest that higher levels of social acknowledgment and self‐disclosure are associated with greater PTG in longer term following traumatic loss.
- Measurements at different time points and different types of social support may play distinct roles in the complex area of promoting PTG after loss.

## 1 Introduction

The traumatic loss of a loved one is one of the most frequently reported and most feared potentially traumatic life events worldwide (Benjet et al. 2016; Wild et al. 2023). Traumatic losses can be defined from two perspectives: on the one hand, based on their objective circumstances, such as murder, suicide, accident, or in the context of war, terrorism, or disaster (Boelen et al. 2019), and on the other hand, based on the subjective perception of the bereaved as particularly sudden, violent, unjust, or preventable (Barlé et al. 2017). The associated burdens on the bereaved are often substantial and can culminate in significantly increased suicide and general mortality rates of around 20% after a loss (Stroebe et al. 2007). If the circumstances of death are sudden and unnatural, the likelihood of various mental disorders also increases significantly (Dyregrov et al. 2003; Kristensen et al. 2014). Traumatic losses typically meet the event criteria for both posttraumatic stress disorder (PTSD)—‘exposure to actual or threatened death, serious injury, or sexual violence, experienced through direct involvement, witnessing, learning it occurred to a close relative/friend’ (APA 2022)—and prolonged grief disorder (PGD)—‘the death of a person close to the bereaved’ (APA 2022). Consequently, both disorders are frequently associated with such losses (Jann et al. 2024). Smith and Ehlers (2021) described this as a ‘dual impact’, where the traumatic event and the event of loss occur simultaneously. While the connection between posttraumatic stress and grief was first linked by Horowitz (1974) as stress‐response syndrome, the aspect of grief was largely neglected for a long time afterward (Neria and Litz 2004). It was only decades later that a significant movement emerged to examine grief processes from a clinical perspective, which has since been referred to by various names: ‘traumatic grief’ (Prigerson et al. 1999), ‘complicated grief’ (CG; Shear et al. 2011), ‘persistent complex bereavement disorder’ (PCBD; APA 2013) and most recently, ‘prolonged grief disorder’ (PGD; APA 2022; WHO 2019). All these diagnostic proposals share the aim of distinguishing maladaptive grief from normal grief processes, with significant overlap in their primary criteria: (a) the loss of a close relative; (b) yearning; (c) cognitive, emotional and behavioural symptoms, such as avoidance behaviour or difficulties in accepting the loss (see APA 2013; APA 2022; Prigerson et al. 1999; Shear et al. 2011; WHO 2019).

However, in addition to the exclusively deficit‐oriented perspectives, there is increasing evidence of potentially positive psychological changes resulting from processing traumatic losses (e.g., Hurst and Kannangara 2024; Levi‐Belz et al. 2021). This phenomenon is often referred to as posttraumatic growth (PTG) and is based on coping‐oriented cognitive and emotional processing, which can be associated with increased appreciation of life, improved interpersonal relationships, the recognition of new life possibilities, increased feelings of personal strength and spiritual development (Tedeschi and Calhoun 2004; Tedeschi and Calhoun 1996). In this sense, PTG implies development beyond the initial level in emotional, cognitive or functional domains (Henson et al. 2021).

In addressing the question of how PTG develops, Tedeschi and Calhoun (1995) originally proposed the functional descriptive model of PTG. Since the beginning of PTG research, they particularly focused on intrapersonal factors such as posttraumatic cognitions, emotional distress and rumination, as well as interpersonal factors like social support and self‐disclosure. Later developed etiological models also maintained this focus as core components for promoting PTG (Joseph et al. 2012). Michael and Cooper (2013) were indeed able to empirically confirm in their systematic review that the three components (a) loss‐related characteristics, (b) cognitive processes and (c) social environment are of great importance. In addition, to the best of our knowledge, the only meta‐analysis to date regarding PTG following traumatic losses confirmed that loss‐related variables, as well as intrapersonal and interpersonal factors, appear to be highly relevant for the prediction of PTG (Levi‐Belz et al. 2021). However, it is noteworthy that the data from the underlying studies were collected only months to years after the losses. In the present study, we aimed to examine these areas for the first time immediately after the death event and again after 6 months. This can aid in better understanding the diversity of individual initial conditions and adaptation processes.

### 1.1 Loss‐Related Factors and PTG

In a systematic review, Hurst and Kannangara (2024) showed that losses are often an initial event for PTG, supporting the assumption that traumatic losses, as a subset of traumatic experiences, provide fertile ground for PTG. Some studies confirmed that bereaved individuals with traumatic losses due to earthquakes (Şenyüz et al. 2021), suicides (Levi‐Belz et al. 2021; Creegan et al. 2024), perinatal loss (Alvarez‐Calle and Chaves 2023) and in mixed samples with violent losses (Milman et al. 2018; Yilmaz and Zara 2016) report higher PTG than those without potentially traumatic death circumstances. There is also evidence that the question of who dies makes a difference for PTG: Several studies have shown that it is not so much the degree of kinship to the deceased, but rather the emotional closeness of the relationship that is decisive for higher PTG symptoms (Chen and Tang 2021; Drapeau et al. 2019; Johnsen and Afgun 2021; Levi‐Belz 2017). In addition, it has been shown that the more time has passed since the death event, the more PTG becomes likely (Michael and Cooper 2013; Levi‐Belz et al. 2021; Yilmaz and Zara 2016). It was found that PTG significantly increased with the months (Yilmaz and Zara 2016) and even years (Levi‐Belz 2015; Drapeau et al. 2019) following the loss. Interestingly, there seems to be a peak in PTG, at least among bereaved individuals after suicide, occurring 9 years post‐loss, after which PTG declines again (Levi‐Belz et al. 2021).

*We expect that higher levels in loss‐related factors such as traumatic circumstances, the perception of death as sudden or violent, a close relationship to the deceased and more time since loss will be positively associated with PTG*.

### 1.2 Intrapersonal Factors of PTG Following Traumatic Loss

Regarding intrapersonal factors, cognitive processes and levels of distress are the focus of etiological models and empirical studies (Joseph et al. 2012; Levi‐Belz et al. 2021; Michael and Cooper 2013; Tedeschi and Calhoun 1995).

The role of cognitive factors and PTG following traumatic loss is particularly interesting regarding the theory of shattered assumptions, according to Janoff‐Bulman (1989), as it is often considered a central precondition for PTG as a cognitive burden (Tedeschi and Calhoun 2004). These considerations are even more important in the context of traumatic losses, as they are among the events that shatter assumptions the most (Wild et al. 2023). Intrapersonal coping with shattered beliefs requires active cognitive engagement with the altered reality, whereby new, coherent images of the world and the self are constructed (Tedeschi and Calhoun 2004). Such negative assumptions about the world and the self are typically summarised and assessed as posttraumatic cognitions (e.g., Foa et al. 1999). Studies examining posttraumatic cognitions in relation to PTG after (traumatic) losses found that maladaptive cognitive coping strategies were not associated with PTG (Levi‐Belz 2015; Moore et al. 2015). These empirical findings are consistent with the assumption that adaptive cognitive processes are crucial for PTG (Tedeschi and Calhoun 2004).

*We expect that lower posttraumatic cognition severity is associated with higher PTG*.

A second intrapersonal component, the level of distress peri‐ and post‐event, is a subject of controversial discussion in context of PTG. Distress has often been measured as symptomatic distress in the form of symptoms of PTSD, anxiety or depression (e.g., Bernard et al. 2022; Pino et al. 2022; Zhen and Zhou 2022). When developing the concept, Tedeschi and Calhoun (2004) already suspected that psychological distress and growth are not opposites but can occur simultaneously. They even postulated that PTG often occurs only when accompanied by significant subjective distress and wrote: ‘The maintenance of the growth experienced may require unpleasant periodic cognitive reminders of what has been lost, so that in an apparently paradoxical way, what has been gained remains in focus’. (Tedeschi et al. 2015, 505). From this, it follows that PTG does not lead to a reduction in psychological distress. Rather, they postulate a high level of distress as a necessary condition for the emergence and maintenance of PTG. Levi‐Belz et al. (2021) showed that PTG can often run parallel to symptoms of PTSD and PGD following suicide loss rather than being mutually exclusive. Research in other areas of traumatic events also supports that retrospectively assessed higher distress during the immediate event is associated with higher PTG (e.g., Greene 2018).

*We expect that higher symptomatic distress is associated with higher PTG*.

### 1.3 Interpersonal Factors of PTG Following Traumatic Loss

With regard to interpersonal factors, studies have indeed categorised the positive influence of self‐disclosure and perceived social support as highly significant for PTG after loss (Alvarez‐Calle and Chaves 2023). The etiological models introduced at the beginning have consistently emphasised that interactive exchange can positively impact posttraumatic cognitions and, consequently, PTG (Joseph et al. 2012; Tedeschi and Calhoun 1995). This is explained by the active confrontation with the loss through storytelling, the fulfilment of attachment theory needs and the opportunity to reconsider the event from different perspectives. Partnership support (Albuquerque et al. 2018; Tian and Solomon 2020) and help from people who have experienced something similar (Tedeschi et al. 2018) are particularly emphasised. Levi‐Belz et al. (2021) identified perceived social support and self‐disclosure as proactive coping strategies as central correlates of PTG following suicide loss based on cross‐sectional and longitudinal studies. However, questions remain regarding other traumatic losses and the association of interpersonal factors with PTG immediately after the loss. The question of whether interpersonal variables are also significantly associated with PTG in other traumatic losses, as well as the timing of these associations, is crucial for clinical practice.

*Based on the literature, we postulate a positive association of interpersonal factors (social support, social acknowledgement*, self*‐disclosure) with PTG*.

### 1.4 Contribution

Despite the apparent connection, there are relatively few studies on the relationship between traumatic losses and PTG (for an overview, see Hurst and Kannangara 2024). In their review of bereaved individuals following suicide loss, Levi‐Belz et al. (2021) revealed a critical lack in the literature regarding studies on other types of traumatic loss. This gap exists despite the likelihood that PTG processes vary depending on the nature of (a) loss‐related characteristics, (b) intrapersonal factors (posttraumatic cognitions, symptomatic distress) and (c) interpersonal factors (social support, social acknowledgment, self‐disclosure). To the best of our knowledge, there are also no studies that have investigated this phenomenology in the first months or even weeks or days after traumatic loss, so this area seems to be completely unexplored. Summarising the introduced hypotheses in each section, we explore the overarching question of which factors within the three domains are associated with PTG after 6 months, at what time points, and in which direction.

In light of etiological assumptions of PTG, the early identification of factors that are positively associated with PTG is crucial, as it enables the development of interventions aimed at specifically enhancing these factors. This can help not only in reducing symptoms but also in promoting resilient outcomes. Therefore, this is of particular relevance for psychosocial crisis intervention as well as for clinical practice in the longer term. The longitudinal design could also reveal more differentiated aspects, especially regarding intrapersonal and interpersonal factors over time. This allows us to examine the complex relationship between both factors over time more precisely, providing initial insights into whether there are critical time windows for promoting intrapersonal or interpersonal factors during which they are associated with PTG.

## 2 Methods

### 2.1 Participants and Procedure

Participants were recruited in cooperation with the emergency counselling services in 18 operational areas in North Rhine‐Westphalia, Germany. These church‐based programs are integrated into the structures of the fire brigade and police and support individuals affected by stressful events. The study was preregistered (registration doi: https://doi.org/10.17605/OSF.IO/A6HQX) and approved by Bielefeld University's ethics committee.

Participants were subtly invited to participate in the study through informational material. Inclusion criteria were the death of a close relative and being at least 18 years old. The survey enabled both paper‐based and online participation via LimeSurvey. Data collection took place between August 2022 and January 2024. A total of 746 questionnaires were distributed; *N* = 135 people took part in the first measurement point immediately after the loss (T1), and *N* = 45 people took part again in the second measurement point after 6 months (T2). A total of nine cases were excluded due to the type of relationship with the deceased person (just acquaintance or stranger), resulting in a final sample of *n* = 36 (T2). In the analysis of a systematic dropout of categorical and continuous variables among participants assessed at T1 and T2, we found a significantly smaller age difference to the deceased as well as higher perceived social support immediately after the loss (cf. Tables S1 and S2).

**FIGURE 1:**
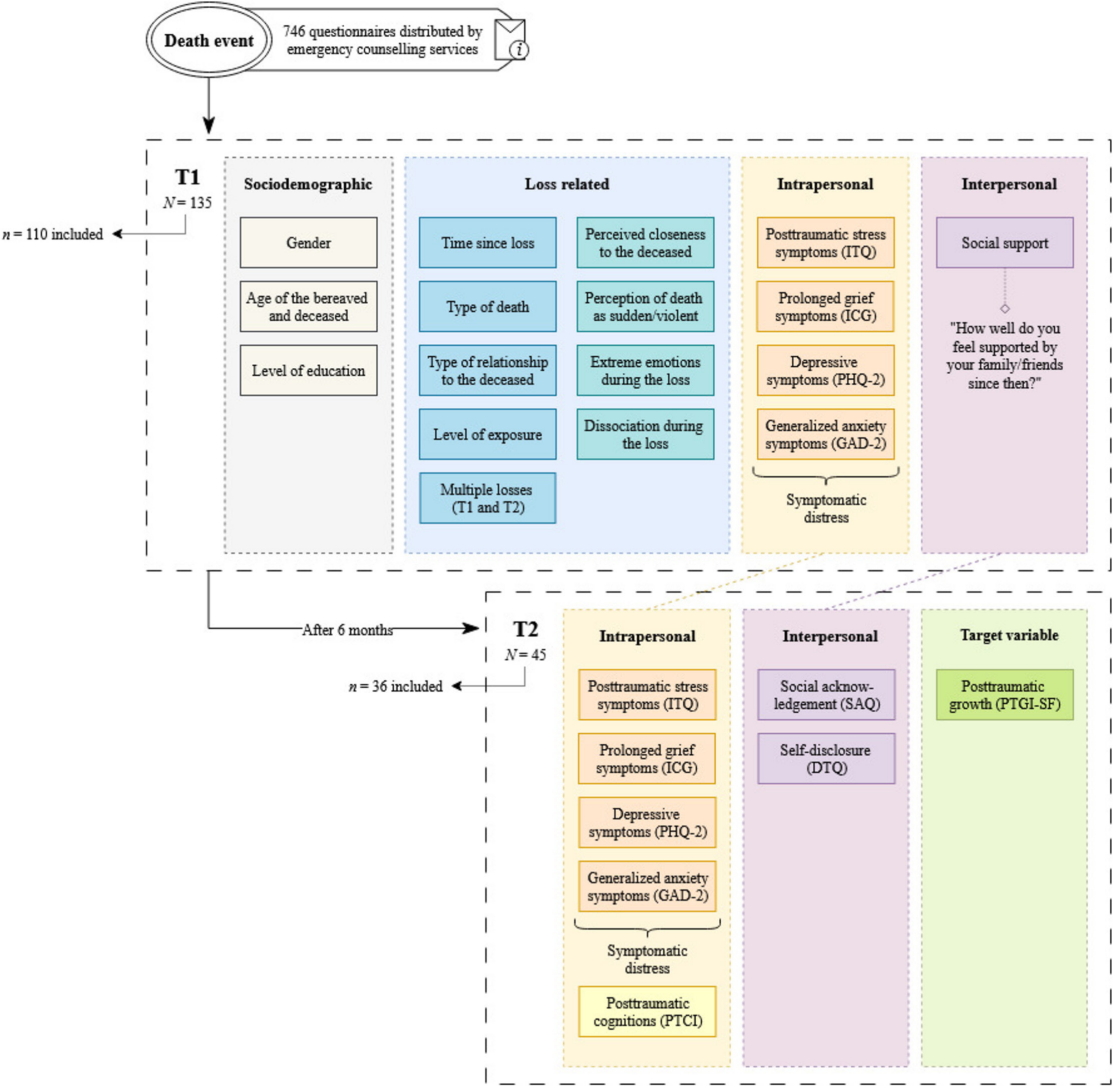
Overview of the study design and measurements. T1 was assessed immediately after the loss and T2 in a follow‐up after 6 months. Inclusion criteria were the death of a close relative and being at least 18 years old. DTQ, Disclosure of Trauma Questionnaire (Müller et al. 2000); GAD‐2, General Anxiety Disorder‐2 (Kroenke et al. 2007); ICG, Inventory of Complicated Grief (Lumbeck et al. 2012); ITQ, International Trauma Questionnaire (Cloitre et al. 2018); PHQ‐2, Patient Health Questionnaire‐2 (Kroenke et al. 2003 PTCI, Posttraumatic Cognitions Inventory (Foa et al. 1999); PTGI‐SF, Posttraumatic Growth Inventory—Short Form (Cann et al. 2010); SAQ, Social Acknowledgement Questionnaire (Maercker and Müller 2004). Measurement details for all variables are described in the ‘Methods’ section.

An overview of the design and the measurements at the respective measurement points is presented in Figure 1.

### 2.2 Measurements

#### 2.2.1 Sociodemographic and Loss‐Related Characteristics (T1)

To record sociodemographic characteristics, we used a purpose‐built questionnaire at the first measurement point, which recorded the age of the bereaved and deceased person and the gender and level of education of the bereaved person. In addition, we recorded loss‐related characteristics, including the time interval since the event, the cause of death, the type of relationship with the deceased person, the level of exposure and any other losses in the previous months. An overview of the characteristics of the variables surveyed can be found in Table 1.

**TABLE 1:** Sociodemographic and loss‐related characteristics of the final sample (*n* = 36).

Sociodemographic characteristics		
	*Mdn*	*SD*
Age	49.00	18.41
	*n*	%
Gender		
Male	15	41.67
Female	21	58.33
Level of education^a^		
No degree	0	0.00
Hauptschulabschluss	7	19.44
Realschulabschluss	10	27.78
Fachhochschulreife	8	22.22
Allgemeine Hochschulreife	6	16.67
University degree	5	13.89

Loss‐related characteristics				
	*Mdn*		*SD*	
Time since loss (hours)	97.00		378.26	
	*n*		%	
Age difference to the deceased				
Younger than the deceased	15		41.67	
Same age	2		5.56	
Older than the deceased	19		52.78	
Relationship to the deceased				
Child	8		22.22	
Spouse	9		25.00	
Parent	3		8.33	
Sibling	2		5.56	
Grandparent	1		2.78	
Other degree of kinship	1		2.78	
Partner	3		8.33	
Friend	9		25.00	
Type of death				
Illness	5		13.89	
Old age	1		2.78	
Unsuccessful resuscitation	7		19.44	
Accident	5		13.89	
Suicide	7		19.44	
Murder	7		19.44	
Other reason	4		11.11	
Level of exposure				
During death	13		36.11	
After death	16		44.44	
None	7		19.44	
	**T1**		**T2**	
**Multiple losses**	***n***	**%**	***n***	**%**
Yes	19	52.78	10	27.78
No	17	47.22	26	72.22

*Note:* For continuous variables, medians (*Mdn*) and standard deviations (*SD*) are reported; for categorical variables, the number of participants (*n*) and corresponding percentages (%) are reported. Total sample size = 36.

^a^ Hauptschulabschluss and Realschulabschluss correspond to secondary school certificates; Fachhochschulreife corresponds to qualification for university of applied sciences admission; Allgemeine Hochschulreife corresponds to qualification for university admission.

As part of the overarching research project, we also recorded potential risk factors for the development of PTSD and PGD, which were also included in our analysis. These include loss‐related variables such as perceived emotional closeness to the deceased person, the perception of death as sudden or violent, the experience of extreme emotions (e.g., fear, helplessness, horror, guilt, or shame) and dissociation during the loss. The assessments used a 7‐point Likert scale from 1 (‘*does not apply at* all’) to 7 (‘*applies very strongly*’).

#### 2.2.2 Intrapersonal Factors (T1 and T2)

Posttraumatic stress symptoms were recorded at both measurement times using the German‐language version of the International Trauma Questionnaire (ITQ; Cloitre et al. 2018). Using the first six items, we recorded the core PTSD symptoms: intrusion, avoidance and hyperarousal. Participants rated their experience on a 5‐point Likert scale from 0 (‘*not at all*’) to 4 (‘*very strongly*’). The present study showed good to high reliability with Cronbach's *α* = 0.80 (T1) and *α* = 0.91 (T2).

CG was measured at both time points using the German version of the Inventory of Complicated Grief (ICG; Lumbeck et al. 2012) based on Prigerson et al. (1995). The questionnaire comprises 19 items on grief reactions on a cognitive, emotional, physiological and behavioural level, rated on a scale from 0 (‘*never*’) to 4 (‘*always*’). Although the ICG items deviate slightly from the current PGD criteria in established nosologies, studies have demonstrated good convergent validity of the ICG with other measures of grief symptoms (Boelen and Lenferink 2020), and even superior accuracy in identifying individuals with maladaptive grief compared with measurements based on the current ICD‐11 and DSM‐5 criteria (Cozza et al. 2016, 2020). Our study showed a high internal consistency of Cronbach's *α* = 0.90 (T1) and *α* = 0.94 (T2).

Depressive symptoms were assessed using the Patient Health Questionnaire‐2 (PHQ‐2; Kroenke et al. 2003) at both measurement times. This instrument comprises two items on the core symptoms of depression: loss of interest and low mood, rated on a scale from 0 (‘*never*’) to 4 (‘*always*’). The present survey also yielded good values for internal consistency with *α* = 0.86 (T1) and *α* = 0.85 (T2).

We assessed generalised anxiety symptoms with the Generalised Anxiety Disorder‐2 (GAD‐2; Kroenke et al. 2007). This short instrument consists of two items on worry control and inner restlessness or anxiety, rated on a scale from 0 (‘*never*’) to 4 (‘*always*’). In our study, Cronbach's *α* = 0.76 (T1) and *α* = 0.90 (T2) were acceptable to high.

To assess posttraumatic cognitions, we used the Posttraumatic Cognitions Inventory (PTCI; Foa et al. 1999) at T2. The questionnaire comprises 37 items on negative beliefs about the self, negative assumptions about the world and self‐blame. The assessment is made on a scale from 1 (‘*strongly disagree*’) to 7 (‘*strongly agree*’). In our sample, Cronbach's *α* = 0.97 (T2) was very high.

#### 2.2.3 Interpersonal Factors (T1 and T2)

We recorded social support at the first measurement point by asking ‘How well do you feel supported by your family/friends since then?’ The assessments were made on a 7‐point Likert scale from 1 (‘*strongly disagree*’) to 7 (‘*strongly agree*’).

We used the Social Acknowledgement Questionnaire (SAQ; Maercker and Müller 2004) at T2 to determine the social recognition and support of those affected after the loss. The questionnaire comprises 16 items that reflect perceived social recognition and support or rejection and stigmatisation. The assessment was based on a 5‐point Likert scale from 1 (‘*strongly disagree*’) to 5 (‘*strongly agree*’). In the present study, the internal consistency of *α* = 0.60 was low.

We recorded self‐disclosure—the communication behaviour about the loss—using the Disclosure of Trauma Questionnaire (DTQ; Müller et al. 2000) at T2. The instrument consists of 34 items, which are divided into three subscales: reasons for or against talking about the event, frequency and intensity of disclosure and emotional reactions during disclosure. The assessment was carried out on a 6‐point Likert scale from 0 (‘*strongly disagree*’) to 5 (‘*strongly agree*’). In the present study, a high internal consistency of *α* = 0.89 was achieved.

#### 2.2.4 PTG (T2)

To assess PTG, we utilised the Posttraumatic Growth Inventory—Short Form (PTGI‐SF; Cann et al. 2010) at T2. This instrument measures personal strength, new possibilities, improved relationships, spiritual growth and appreciation for life, with two items for each domain. Participants were asked whether they had experienced changes in these areas in the 6 months following their loss. They could respond using a 6‐point Likert scale ranging from 0 (‘*not at all*’) to 5 (‘*very much*’). The PTGI‐SF demonstrated high internal consistency in the present study, with a Cronbach's alpha of 0.93.

### 2.3 Statistical Analyses

We performed the statistical analysis using R (version 4.4.2; R Core Team 2024) and RStudio (version 2024.12.0.467; Posit Team 2024). We replaced missing values (1.35%) with Multiple Imputation by Chained Equations (MICE) using the R package mice (van Buuren and Groothuis‐Oudshoorn 2011), creating five complete data sets according to Rubin (1987).

First, we performed descriptive analyses to capture sociodemographic and loss‐related characteristics. We then used inferential statistical analyses with a significance level of *α* = 0.05. The associations between PTG and relevant variables were mainly analysed using Pearson or Spearman correlation analyses. For group comparisons, we applied Student's *t*‐tests. For variables that violated the normal distribution assumption, we conducted Mann–Whitney *U* tests or Kruskal–Wallis tests.

Second, given that the sample size was too small to include all listed factors in the regression analyses while maintaining sufficient statistical power, we made specific methodological decisions at this point. As sociodemographic and loss‐related variables did not show significant associations with PTG and were examined primarily in an exploratory manner, we focused on intrapersonal and interpersonal variables in the next step, due to the etiological and empirical relevance of these variables in understanding PTG.

To investigate the predictive value of intrapersonal and interpersonal variables on PTG, we performed multiple linear regression analyses (T1 → T2 and T2 → T2). We combined the psychological stress measures (ITQ, ICG, PHQ‐2, GAD‐2) per measurement time point in a *z*‐standardised manner into an overall variable ‘Symptomatic Distress’, based on content considerations and high intercorrelations (T1: 0.55 to 0.81; T2: 0.61 to 0.84). We modelled all predictors simultaneously to determine their specific contribution, although we did not consider additional covariates due to the small sample size. A post hoc power analysis with G*Power (version 3.1.9.7, Faul et al. 2007) yielded a test power of 0.75 for both models with four predictors, an assumed effect size of *f* ^2^ = 0.35, a significance level of *α* = 0.05 and a sample size of *n* = 36. We conducted the regression analyses separately for each of the five imputed data sets and pooled the results according to Rubin (1987). Before estimating the model, we checked the statistical assumptions. To minimise possible biases due to outliers and heteroscedasticity, we used robust regressions with the MM estimator according to Yohai (1987). We pooled all analyses according to the method of Rubin (1987).

## 3 Results

### 3.1 Sociodemographic and Loss‐Related Characteristics

Table 2 summarises the sociodemographic and loss‐related characteristics, along with the results of group comparisons examining differences in the level of PTG. The sample is heterogeneous in terms of gender, different age groups and educational level. The high level of symptomatic distress in PTSD, PGD, depression and general anxiety is striking (cf. Table S3). The participants were highly affected, as all of the deceased were either close friends or family members (*n* = 36, 100%), most participants were confronted with unnatural types of deaths (*n* = 30, 83%) and exposed to the dead body during or after the event (*n* = 29, 81%). All participants fulfil at least one characteristic of traumatic losses according to the definitions listed at the beginning.

**TABLE 2:** Statistical group comparisons of PTG across sociodemographic and loss‐related characteristics.

	Group descriptives (PTG)					Student's *t*‐test					
Grouping variable	Group	*n*	Mean	SD		*t*	*df*	*p*	Cohen's *d*	95% CI	
										Lower	Upper
Level of education^a^	Lower	17	15.77	11.06							
	Higher	19	17.11	13.68		0.32	34	0.750	0.11	−0.55	0.76
Sudden/violent	Lower	20	14.75	12.80							
	Higher	16	18.63	11.82		0.93	34	0.357	0.31	−0.35	0.97
Dissociation	Lower	25	17.20	13.17							
	Higher	11	14.82	10.66		−0.53	34	0.601	−0.19	−0.90	0.52
						**Krusal–Wallis test**					
	**Group**	***n***	**Mean**	***SD***		***H***	***df***	***p***	**Rank** ***η*** ^***2***^	**95% CI**	
										**Lower**	**Upper**
Level of exposure	During death	13	21.23	11.31							
	After death	16	12.69	12.02							
	None	7	16.29	13.65		5.25	2	0.073	0.10	0.00	0.44
						**Mann–Whitney *U* test**					
	**Group**	***n***	**Mean**	***SD***	**Mean rank**	***U***	***p***		**Rank‐biserial correlation**	**95% CI**	
										**Lower**	**Upper**
Gender	Male	15	13.47	12.72	15.87						
	Female	21	18.62	11.93	20.38	118.00	0.210		−0.25	−0.57	0.13
Age of the bereaved	Lower	18	13.50	11.85	15.83						
	Higher	18	19.44	12.45	21.17	210.00	0.132		0.30	−0.08	0.60
Type of death^b^	Natural	15	15.13	13.56	16.80						
	Unnatural	21	17.43	11.66	19.71	132.00	0.422		−0.16	−0.50	0.22
Previous losses (T1)^c^	Yes	19	12.84	10.10	15.79						
	No	17	20.53	13.64	21.53	110.00	0.106		−0.32	−0.61	0.05
Subsequent losses (T2)^d^	Yes	10	15.70	10.64	18.05						
	No	26	16.77	13.14	18.67	125.50	0.888		−0.04	−0.43	0.37
Extreme emotionse^e^	Lower	19	17.74	12.81	19.55						
	Higher	17	15.06	12.04	17.32	141.50	0.536		−0.12	−0.47	0.25
Closeness to the deceased	Lower	9	20.44	13.78	21.94						
	Higher	27	15.15	11.82	17.35	90.50	0.265		−0.26	−0.61	0.18

*Note: n* = group size; *SD* = standard deviation. *t*, *U*, and *H* represent the test statistics of the Student's *t*‐test, Mann–Whitney *U* test, and Kruskal–Wallis test, respectively. *df* = degrees of freedom; *p* = significance level. Effect size is given by Cohen's *d* for the Student's *t*‐test, rank *η* ^2^ *f*or the Kruskal–Wallis test, and rank‐biserial correlation for the Mann–Whitney *U* test. 95% CI = 95% confidence interval of the respective effect size. Likert items (7‐point scale from 1 (‘does not apply at all’) to 7 (‘applies very strongly’)) and continuous variables were dichotomised into “lower” and “higher” groups based on a median split.

^a^ Lower = secondary school certificate; higher = qualification for university of applied sciences admission, qualification for university admission, or university degree.

^b^ Natural = illness, old age, unsuccessful resuscitation; unnatural = accident, suicide, murder.

^c^ Assessed immediately after the loss.

^d^ Assessed 6 months after the loss.

^e^ Involves emotions such as fear, helplessness, horror, guilt, or shame.

We found no group differences in PTG after 6 months in relation to age, gender and educational level. We also found no differences in PTG when considering the type of death, suddenness and level of violence of death, level of exposure, dissociation (depersonalisation, derealisation) during the event, emotions (e.g., fear, helplessness, horror, guilt or shame), closeness to the deceased, multiple losses due to previous losses or subsequent losses.

### 3.2 Immediate Intrapersonal and Interpersonal Predictors of PTG

In a direct comparison through regression analysis, immediately perceived social support did not predict later PTG, *β* = 0.30*, p* = 0.199 (Table 3). However, lower acute symptomatic distress is associated with later PTG, *β = −0*.36*, p =* 0.019. This contradicts our hypothesis about the association of high symptomatic distress with PTG. The variance explained is 17% (adjusted *R* ^2^ = 0.17).

**TABLE 3:** Results of the robust regression analysis predicting PTG at T2 based on intrapersonal and interpersonal factors at T1 (T1 → T2).

	PTG *(T1* → *T2)*					
	*b*	*β*	*SE*	*t*	*df*	*p*
Social support	2.58	0.30	1.97	1.31	31.16	0.199
Symptomatic distress^a^	−5.12	−0.36	2.07	−2.48	31.10	0.019
*R* ^2^	0.17					

*Notes:* T1 was assessed immediately after the loss; T2 was assessed 6 months after the loss. *b =* unstandardised coefficient; *β* = standardised coefficient; *SE* = standard error; *t* = test statistic; *df* = degrees of freedom; *p* = significance level; *R* ^2^ = adjusted coefficient of determination.

^a^ Variable created by combining the psychological stress measures (ITQ; Cloitre et al. 2018; ICG; Lumbeck et al. 2012; PHQ‐2; Kroenke et al. 2003; GAD‐2; Kroenke et al. 2007) at T1 in a z‐standardised manner.

### 3.3 Long‐Term Intrapersonal and Interpersonal Predictors of PTG

Table 4 shows that both interpersonal inventories SAQ (*β* = 0.64*, p* < 0.001), and DTQ (*β* = 0.67*, p* = 0.011), are significantly associated with PTG after 6 months. In contrast, intrapersonal variables in the form of symptomatic distress (ITQ, ICG, GAD‐2, PHQ‐2) and posttraumatic cognitions showed no more significant correlations with PTG. The variance explained for PTG is 37% (adjusted *R* ^2^ = 0.37).

**TABLE 4:** Results of the robust regression analysis predicting PTG at T2 based on intrapersonal and interpersonal factors at T2 (T2 → T2).

	PTG *(T2* → *T2)*					
	*b*	*β*	*SE*	*t*	*df*	*p*
Social acknowledgement^a^	0.81	0.64	0.21	3.82	28.54	< 0.001
Self‐disclosure^b^	0.33	0.67	0.12	2.71	29.02	0.011
Symptomatic distress^c^	−2.86	−0.21	4.91	−0.58	28.69	0.566
Posttraumatic cognitions^d^	−0.04	−0.14	0.09	−0.47	28.22	0.642
*R* ^2^	0.37					

*Note:* T2 was assessed 6 months after the loss. *b =* unstandardised coefficient; *β* = standardised coefficient; *SE* = standard error; *t* = test statistic; *df* = degrees of freedom; *p* = significance level; *R* ^2^ = adjusted coefficient of determination.

^a^ Measured with the Social Acknowledgement Questionnaire (SAQ; Maercker and Müller 2004).

^b^ Measured with the Disclosure of Trauma Questionnaire (DTQ; Müller et al. 2000).

^c^ Variable created by combining the psychological stress measures (ITQ; Cloitre et al. 2018; ICG; Lumbeck et al. 2012; PHQ‐2; Kroenke et al. 2003; GAD‐2; Kroenke et al. 2007) at T2 in a z‐standardised manner.

^d^ Measured with the Posttraumatic Cognitions Inventory (PTCI; Foa et al. 1999).

## 4 Discussion

This is the first study to investigate factors promoting PTG immediately after a traumatic loss and 6 months later. A particular focus is on intrapersonal and interpersonal variables, which are central components in the most common etiological models of PTG. The sample shows a high degree of involvement through close relationships with the deceased, often sudden or violent death events and often direct exposure by seeing the corpse. In general, loss‐related and sociodemographic variables appear to be less relevant for PTG. We found evidence that lower immediate symptomatic distress predicts later PTG. This contradicts the common assumption that high symptomatic distress is a prerequisite for later PTG. While immediately perceived social support is less relevant, social acknowledgment and self‐disclosure appear to be more relevant 6 months later, whereas the importance of intrapersonal variables such as posttraumatic cognitions and symptomatic distress has less relevance then. Intrapersonal and interpersonal factors as central components of etiological models show relevance but at different time points, highlighting the complexity of psychopathology and PTG as well as their progression. This underscores the urgent need for further research at the immediate time point of PTG following traumatic events in general, as well as particularly traumatic losses.

Lower symptomatic distress immediately after the experienced loss was associated with a higher level of later PTG. This contradicts the hypothesis derived from the literature that a high degree of symptomatic distress is necessary for PTG (e.g., Tedeschi et al. 1995, 2015; Tedeschi and Calhoun 2004). The results also contradict the frequently held assumption that high symptomatology and PTG occur simultaneously (Pino et al. 2022; El Khoury‐Malhame et al. 2023). One explanation could be that the relationship between distress and PTG is not linear. There are findings suggesting that a moderate degree of distress is associated with the highest probability of PTG, indicating an inverted U‐shaped relationship between distress and PTG (cf. Milman and Neimeyer 2020). Distress, therefore, plays a dual role: On the one hand, it is a prerequisite for triggering inner change; on the other hand, it must be reduced to a manageable level to enable a constructive confrontation with the event. Given that the present sample shows very high symptomatic distress and levels of exposure, this might have been too high for PTG. There could be a symptomatic threshold beyond which PTG becomes significantly hindered. It is also possible that high posttraumatic symptoms in terms of intrusion, avoidance, hypervigilance and the persistent perception of threat in the ‘here and now’ block proactive, positive processing (Wild et al. 2023). Since our research design does not allow a conclusive examination of the postulated inverse U‐shaped relationship between distress and PTG, future research should further explore this area immediately after traumatic losses.

While social support appears to be less important immediately after the loss, social acknowledgment and self‐disclosure become more significant in the longitudinal course. These interpersonal factors have already been shown in previous studies and etiological models to be central for PTG after loss (Tedeschi and Calhoun 2004; Levi‐Belz et al. 2021). As an explanation, support for positive cognitive processing and protection from isolation, especially after stigmatised losses such as suicide, is emphasised (Levi‐Belz 2015; Levi‐Belz et al. 2021). Aksu (2024) also showed that self‐disclosure supports deliberate rumination, whereby traumatic experiences are reinterpreted and changes in perspective in the sense of PTG can occur. The relevance of both factors is particularly interesting, as it can be assumed that proactive high self‐disclosure as a coping strategy is also associated with higher social acknowledgment and social support as well as less social isolation, both acutely and in the long term (Alvarez‐Calle and Chaves 2023). All three factors may therefore be mutually dependent and may even be a necessary initial condition for each other. The fact that social support did not emerge as an acute promotive factor could therefore be an expression of the possibility that we recorded it too unspecifically with just one item for T1. In addition to the significance of timing, future research should delve deeper into differentiating interpersonal aspects. For example, distinctions could be made between proactively utilised social structures, such as through self‐disclosure, and passively perceived social acknowledgment and support. Alternatively, distinctions could be drawn between emotional and institutional support following traumatic losses. This differentiation is crucial for gaining more clarity in this important field to improve support for the bereaved.

### 4.1 Strengths and Limitations

The sample has unique characteristics due to its affectedness and time of measurement and is broadly distributed in sociodemographic variables. However, there are some limitations to consider. Although this is a longitudinal study that starts directly after traumatic losses, it was not possible to observe the course of PTG over multiple measurement points or longer periods. Additionally, the small sample size and the acute survey setting led to a reduction in the variables collected and included in the analyses, particularly regarding the measurement of intrapersonal and interpersonal variables, which we only collected superficially immediately after the loss for economic and ethical reasons. Third variables could have remained unconsidered. Due to the moderate power, while significant results are robust, nonsignificant results could be false negatives. In this context, we could not test for moderation or mediation effects due to the small sample. Despite the longitudinal design, no causal conclusions are indicated. Another limitation is the high dropout rate (67%), which is why we decided to analyse only the full data cases. Participants who took part in both measurement points systematically reported higher social support than those who participated only at T1. Because higher levels of self‐disclosure and social acknowledgement were closely associated with social support, we assume that these participants also exhibit a greater willingness to report on the loss in written form.

### 4.2 Implications for Research and Practice

Our study provides indications that the immediate period following traumatic loss has distinct characteristics that have so far gone unnoticed. This is illustrated, for example, by the refutation of the hypothesis that high intrapersonal distress is necessary for later PTG. Additionally, some studies have shown that PTG can continue to change over many years (Levi‐Belz 2023). Consequently, there is a need for more longitudinal studies that start immediately and collect data over several years. The present study also proves that data collection is feasible even in sensitive contexts immediately after traumatic losses. It should encourage scientists to conduct research in this important field, as many aspects remain largely unexplored (Jann et al. 2024). It is expected that such complex situations are subject to correspondingly complex relationships concerning PTG, so further and larger studies should investigate the relationships between loss‐related, intrapersonal and interpersonal factors and PTG. Considering cognitive models and the present findings, it would be particularly interesting to see whether interpersonal variables, due to their attributed effects on deliberate rumination, perspective change and meaning‐making (Aksu 2024; Milman and Neimeyer 2020), might serve as mediators between intrapersonal factors and PTG. The assessment of the research field regarding immediate data collection and traumatic losses highlights the importance of further research in these areas concerning PTG, as this setting has been under‐researched to date.

In terms of practical implications, the results may be of particular relevance to psychosocial crisis intervention teams and other actors who directly interact with individuals affected by traumatic loss. The findings suggest that reduced acute symptomatic distress may promote PTG. Furthermore, the findings encourage focusing on social acknowledgment and supporting self‐disclosure in the longer term. Alongside institutional support (e.g., psychosocial crisis intervention, self‐help groups, professionals, funeral services), which is particularly crucial for socially marginalised individuals, the involvement of family and friends is essential for bereaved individuals with a supportive social environment. Some studies have shown that support from close people is more important than institutional or formal support (Aoun et al. 2018; Alvarez‐Calle and Chaves 2023). If possible, family and friends should be directly empowered to form a socially sustainable network (Hobfoll et al. 2007). At the same time, it is known from other research reviews following traumatic losses that lack of social support is also the greatest risk factor for psychopathological outcomes, especially PTSD (Jann et al. 2024). Overall, social support may play an important role in simultaneously promoting PTG and reducing PTSD and PGD.

## 5 Conclusions

This study is the first to provide important insights into the factors promoting PTG immediately after traumatic loss. Our findings provide preliminary indications that the general assumption that PTG necessitates high acute symptomatic distress may not apply, at least in the context of traumatic loss. Additionally, the findings suggest that social support should be maintained for an extended period after traumatic losses and that social support itself encompasses various facets that may differ regarding their relevance in promoting PTG. Generally, it can be inferred that the initial hours to days following traumatic losses, as well as the subsequent weeks, have not yet been thoroughly investigated in the existing body of research. The present results suggest that there may be unique dynamics at play during these early periods compared with later stages. Further research with larger samples and more extended measurement periods is urgently needed to better understand this complex area. Such data could yield relevant findings that are significant both acutely for psychosocial crisis intervention and in clinical practice to promote positive aspects after traumatic loss.

## Ethics Statement

The project was reviewed by the Ethics Committee of Bielefeld University.

## Consent

All participants provided written informed consent, which included a declaration on consent and data protection. This declaration outlined the objectives and procedures of the study, as well as detailed information regarding the processing of personal and sensitive data.

## Conflicts of Interest

The authors declare no conflicts of interest.

## Supporting information


**Data S1:** Table S1. Selective dropout analysis: Comparison of categorical T1 variables between participants only assessed at T1 (dropouts) and those assessed at both T1 and T2 (completers).
**Table S2:** Selective dropout analysis: Comparison of continuous T1 variables between participants only assessed at T1 (dropouts) and those assessed at both T1 and T2 (completers).
**Table S3:** Medians, standard deviations and correlations (with confidence intervals) between PTG and negative psychological stress measures at the first (T1) and second (T2) measurement point.

## Data Availability

Data available on request from the authors.
