# Supplementary material for: Intrapersonal and Interpersonal Factors Promoting Posttraumatic Growth: A Longitudinal Study Immediately After Traumatic Loss
Source: Clin Psychol Psychother. 2025 Nov 19;32(6):e70174. doi: 10.1002/cpp.70174 (PMC12628115; doi:10.1002/cpp.70174)
Supplement: Supplementary file 1 — Data S1: Table S1. Selective dropout analysis: Comparison of categorical T1 variables between participants only assessed at T1 (dropouts) and those assessed at both T1 and T2 (completers). Table S2: Selective dropout analysis: Comparison of continuous T1 variables between participants only assessed at T1 (dropouts) and those assessed at both T1 and T2 (completers). Table S3: Medians, standard deviations and correlations (with confidence intervals) between PTG and negative psychological stress measures at the first (T1) and second (T2) measurement point. [file CPP-32-e70174-s001.docx]

**SUPPLEMENTARY MATERIAL**

**Table A1**

*Selective dropout analysis: Comparison of categorical T1 variables between participants only assessed at T1 (dropouts) and those assessed at both T1 and T2 (completers)*

| **Categorical T1 variable** | **Dropouts (*n* = 74)** | | **Completers (*n* = 36)** | | **Chi-squared test** | | |  |
| --- | --- | --- | --- | --- | --- | --- | --- | --- |
|  | *n* | % | *n* | % | *X²* | *df* | *p* | *φ*/Cramer’s *V* |
|  |  |  |  |  |  |  |  |  |
| **Gender** |  |  |  |  | 0.06 | 1 | .803 | .02 |
| Male | 29 | 39.19 | 15 | 41.67 |  |  |  |  |
| Female | 45 | 60.81 | 21 | 58.33 |  |  |  |  |
| **Level of education ^a^** |  |  |  |  | 0.60 | 4 | .963 | .07 |
| No degree | 0 | 0.00 | 0 | 0.00 |  |  |  |  |
| Hauptschulabschluss | 15 | 20.27 | 7 | 19.44 |  |  |  |  |
| Realschulabschluss | 22 | 29.73 | 10 | 27.78 |  |  |  |  |
| Fachhochschulreife | 12 | 16.22 | 8 | 22.22 |  |  |  |  |
| Allgemeine Hochschulreife | 14 | 18.92 | 6 | 16.67 |  |  |  |  |
| University degree | 11 | 14.87 | 5 | 13.89 |  |  |  |  |
| **Relationship to the deceased ^b^** |  |  |  |  | 1.43 | 2 | .488 | .11 |
| Family member | 31 | 41.89 | 15 | 41.67 |  |  |  |  |
| Partner/spouse | 31 | 41.89 | 12 | 33.33 |  |  |  |  |
| Friend | 12 | 16.22 | 9 | 25.00 |  |  |  |  |
| **Type of death ^c^** |  |  |  |  | 2.25 | 3 | .523 | .14 |
| Natural | 40 | 54.80 | 15 | 41.67 |  |  |  |  |
| Accident/other external influence | 12 | 16.44 | 7 | 19.44 |  |  |  |  |
| Suicide | 13 | 17.81 | 7 | 19.44 |  |  |  |  |
| Murder/violent action | 8 | 10.96 | 7 | 19.44 |  |  |  |  |
| **Level of exposure** |  |  |  |  | 0.23 | 2 | .893 | .05 |
| During death | 27 | 36.49 | 13 | 36.11 |  |  |  |  |
| After death | 30 | 40.54 | 16 | 44.44 |  |  |  |  |
| None | 17 | 22.97 | 7 | 19.44 |  |  |  |  |
| **Previous losses** |  |  |  |  | 5.50 | 1 | .019 | .22 |
| Yes | 22 | 29.73 | 19 | 52.78 |  |  |  |  |
| No | 52 | 70.27 | 17 | 47.22 |  |  |  |  |

*Notes*. T1 was assessed immediately after the loss; T2 was assessed six months after the loss. *n* = number of participants; *%* = percentage of participants; *X²* = test statistic of the chi-squared test; *df* = degrees of freedom; *p* = significance level. Effect size is given by *φ* for 2 × 2 contingency tables and Cramer’s *V* for larger contingency tables. Of the initial 135 participants, some were excluded due to their relationship with the deceased (acquaintance, stranger, or unknown), resulting in a sample of *n* = 110 (74 dropouts, 36 completers). Participants with missing data were excluded per analysis. For each variable, original categories were used whenever each category had an expected count of ≥ 5. As some variables were dichotomized for group comparisons of PTG (see Table 2), the selective dropout analysis was also conducted using the dichotomized versions. These analyses yielded no significant results and are not shown in the table.

^a^ Hauptschulabschluss and Realschulabschluss correspond to secondary school certificates; Fachhochschulreife = qualification for university of applied sciences admission; Allgemeine Hochschulreife = qualification for university admission. ^b^ Some original categories with expected counts < 5 were combined: child, parent, sibling, grandparent, and other relative into the category family member, and partner and spouse were combined into a single category.

^c^ Some original categories with expected counts < 5 were combined: natural = illness, old age, unsuccessful resuscitation, and selected cases from the category “other reason” (sudden infant death syndromes, sudden cardiac death, heart attack, stroke, and unknown); accident/other external influence = accident and selected “other reason” cases (drug-related death, COVID-19 vaccination, and brain hemorrhage three weeks after an accident); murder/violent action = murder and selected “other reason” cases (fight and skull-base fracture after conflict).

**Table A2**

*Selective dropout analysis: Comparison of continuous T1 variables between participants only assessed at T1 (dropouts) and those assessed at both T1 and T2 (completers)*

| **Continuous T1 variable** | **Group descriptives** | | | | | **Student’s t-test** | | | | | |
| --- | --- | --- | --- | --- | --- | --- | --- | --- | --- | --- | --- |
|  | Group | *n* | Mean | *SD* | | *t* | *df* | *p* | Cohen’s *d* | 95% CI | |
|  |  |  |  |  |  |  |  |  |  | Lower | Upper |
|  |  |  |  |  | |  |  |  |  |  |  |
| **ICG ^a^** | Dropouts | 71 | 35.38 | 15.24 | |  |  |  |  |  |  |
|  | Completers | 34 | 36.24 | 15.31 | | 0.27 | 103 | .789 | 0.06 | -0.35 | 0.47 |
|  | | | | | | **Mann-Whitney U test** | | | | | |
|  | Group | *n* | Mean | *SD* | Mean rank | *U* | *p* | | Rank-biserial correlation | 95% CI | |
|  |  |  |  |  |  |  |  |  |  | Lower | Upper |
|  |  |  |  |  |  |  |  | |  |  |  |
| **Age of the bereaved** | Dropouts | 74 | 39.89 | 17.55 | 51.82 |  |  | |  |  |  |
|  | Completers | 36 | 46.11 | 18.41 | 63.06 | 1604.00 | .084 | | .20 | -.02 | .41 |
| **Age difference to the deceased ^b^** | Dropouts | 74 | -8.53 | 19.22 | 50.35 |  |  | |  |  |  |
|  | Completers | 36 | 1.72 | 19.29 | 66.08 | 1713.00 | .015 | | .29 | .06 | .48 |
| **Extreme emotions** | Dropouts | 73 | 5.81 | 1.70 | 55.81 |  |  | |  |  |  |
|  | Completers | 36 | 5.83 | 1.54 | 53.36 | 1255.00 | .681 | | -.05 | -.27 | .18 |
| **Closeness to the deceased** | Dropouts | 74 | 6.04 | 1.23 | 53.51 |  |  | |  |  |  |
|  | Completers | 36 | 6.17 | 1.28 | 59.58 | 1479.00 | .312 | | .11 | -.12 | .33 |
| **Sudden / violent** | Dropouts | 73 | 9.84 | 3.47 | 51.32 |  |  | |  |  |  |
|  | Completers | 35 | 11.03 | 2.94 | 61.14 | 1510.00 | .124 | | .18 | -.05 | .40 |
| **Dissociation during loss** | Dropouts | 71 | 9.27 | 3.67 | 53.50 |  |  | |  |  |  |
|  | Completers | 36 | 9.47 | 3.60 | 54.99 | 1313.50 | .816 | | .03 | -.20 | .26 |
| **Social support** | Dropouts | 74 | 5.22 | 1.19 | 50.41 |  |  | |  |  |  |
|  | Completers | 36 | 5.69 | 1.45 | 65.97 | 1709.00 | .013 | | .28 | .06 | .48 |
| **ITQ ^d^** | Dropouts | 72 | 11.64 | 7.21 | 54.09 |  |  | |  |  |  |
|  | Completers | 35 | 11.63 | 5.99 | 53.81 | 1253.50 | .968 | | -.01 | -.24 | .23 |
| **GAD-2 ^e^** | Dropouts | 73 | 4.27 | 2.18 | 50.51 |  |  | |  |  |  |
|  | Completers | 35 | 5.09 | 2.12 | 62.83 | 1569.00 | .054 | | .23 | -.001 | .44 |
| **PHQ-2 ^f^** | Dropouts | 72 | 5.07 | 2.33 | 52.24 |  |  | |  |  |  |
|  | Completers | 36 | 5.58 | 2.23 | 59.03 | 1459.00 | .283 | | .13 | -.11 | .34 |
| **Symptomatic distress ^g^** | Dropouts | 70 | -0.06 | 0.92 | 50.64 |  |  | |  |  |  |
|  | Completers | 33 | 0.10 | 0.81 | 54.88 | 1250.00 | .504 | | .08 | -.16 | .31 |

*Notes.* T1 was assessed immediately after the loss; T2 was assessed six months after the loss. For variables that violated the assumptions of the Student’s t-test, Mann-Whitney U tests were conducted. *n* = group size; *SD* = standard deviation. *t* and *U* represent the test statistics of the Student’s t-test and Mann-Whitney U test, respectively. *df* = degrees of freedom; *p* = significance level. Effect size is given by Cohen’s *d* for the Student‘s t-test and rank-biserial correlation for the Mann-Whitney U test. 95% CI = 95% confidence interval of the respective effect size. Of the initial 135 participants, some were excluded due to their relationship with the deceased (acquaintance, stranger, or unknown), resulting in a sample of *n* = 110 (74 dropouts, 36 completers). Participants with missing data were excluded per analysis. Measurement details for all variables are described in the Methods section.

^a^ Inventory of Complicated Grief (Lumbeck et al., 2012). ^b^ A positive value of the variable indicates that the bereaved person was older than the deceased, and vice versa. ^c^ Involves emotions such as fear, helplessness, horror, guilt, or shame. ^d^ International Trauma Questionnaire (Cloitre et al., 2018). ^e^ General Anxiety Disorder-2 (Kroenke et al., 2007).

^f^ Patient Health Questionnaire-2 (Kroenke et al., 2003). ^g^ Variable created by combining the psychological stress measures (ICG; Lumbeck et al., 2012; ITQ; Cloitre et al., 2018; GAD-2; Kroenke et al., 2007; PHQ-2; Kroenke et al., 2003) at T1 in a z-standardized manner.

**Table B**

*Medians, standard deviations, and correlations (with confidence intervals) between PTG and negative psychological stress measures at the first (T1) and second (T2) measurement point*

|  | *(T1* → *T2)* | | |  | *(T2* → *T2)* | | |
| --- | --- | --- | --- | --- | --- | --- | --- |
| **Variable** | *Mdn* | *SD* | *r / ρ* |  | *Mdn* | *SD* | *ρ* |
|  |  |  |  |  |  |  |  |
| ICG | 37.00 | 15.14 | -.40* *^r^* |  | 24.50 | 16.84 | -.19 |
|  |  |  | [-.65, -.09] |  |  |  | [-.49, -.15] |
|  |  |  |  |  |  |  |  |
| ITQ | 12.00 | 6.13 | -.27 *^r^* |  | 4.50 | 6.21 | -.07 |
|  |  |  | [-.55, .07] |  |  |  | [-.39, .26] |
|  |  |  |  |  |  |  |  |
| PHQ-2 | 6.00 | 2.23 | -.39* |  | 2.00 | 2.50 | -.20 |
|  |  |  | [-.63, -.07] |  |  |  | [-.50, .13] |
|  |  |  |  |  |  |  |  |
| GAD-2 | 5.00 | 2.20 | -.28 |  | 2.00 | 2.53 | -.16 |
|  |  |  | [-.55, .06] |  |  |  | [-.46, .18] |
|  |  |  |  |  |  |  |  |

*Notes.* T1 was assessed immediately after the loss; T2 was assessed six months after the loss. *Mdn* = median; *SD* = standard deviation. Pearson’s correlation (*r*) was used exclusively for correlations marked with *^r^*, as these were the only correlations for which the associated variables met the necessary assumptions (e.g., normality and absence of outliers); all other correlations are based on Spearman’s rank correlation (*ρ*). ICG = Inventory of Complicated Grief (Lumbeck et al., 2012); ITQ = International Trauma Questionnaire (Cloitre et al., 2018); GAD-2 = General Anxiety Disorder-2 (Kroenke et al., 2007); PHQ-2 = Patient Health Questionnaire-2 (Kroenke et al., 2003).

* indicates *p* < .05.
